# What is the difference in morphologic features of the lumbar vertebrae between Caucasian and Taiwanese subjects? A CT-based study: implications of pedicle screw placement via Roy-Camille or Weinstein method

**DOI:** 10.1186/s12891-019-2602-4

**Published:** 2019-05-25

**Authors:** Hsi-Hsien Lin, Jung-Pan Wang, Cheng-Li Lin, Yu-Cheng Yao, Shih-Tien Wang, Ming-Chau Chang, Po-Hsin Chou

**Affiliations:** 1 Department of Orthopedics & Traumatology, Taipei Veterans General Hospital, No. 201, Sec. 2, Shipai Rd., Beitou District, Taipei City, 11217 Taiwan, Republic of China; 2 School of Medicine, National Yang-Ming University, No. 201, Sec. 2, Shipai Rd., Beitou District, Taipei City, 11217 Taiwan, Republic of China; 3 Department of Orthopaedic Surgery, National Cheng Kung University Hospital, College of Medicine, National Cheng Kung University, Tainan, Taiwan, Republic of China

**Keywords:** Pedicle screws, Racial difference, Anatomic study, Roy-Camille method, Weinstein method

## Abstract

**Background:**

Safe placement of pedicle screws without jeopardizing neurovascular structures medially and anteriorly is important during spine surgery. Inferior breach of pedicle is also dangerous due to low margin of error. Lumbar morphology and identical pedicle orientation at L1 to L5 shown on CT scan of young Taiwanese patients (90 patients) were analyzed and compared with findings reported for Caucasian subjects.

**Methods:**

Previously reported techniques were employed to quantitatively elucidate the parameters regarding lumbar morphology and identical pedicle orientation at each vertebra. The parameters for pedicle angle (PA), pedicle diameter (PD), pedicle axis distance (PAD), midline axis distance (MAD), transverse pedicle axis distance (TPAD) and transverse intertangential angle (TITA) were measured.

**Results:**

Taiwanese subjects had different PA, PD, PAD, MAD at L1 to L5 and TITA at L3 to L5 compared with Caucasian subjects. L5 had the most convergent pedicle axis, the widest PD and the shortest antero-posterior axis morphology.

**Conclusions:**

This study provides detailed information for identifying pedicle orientation during pedicle screw placement and elucidate racial differences in lumbar morphology and pedicle orientation between Taiwanese and Caucasian populations.

## Background

Posterior approaches with transpedicular pedicle screw placement for lumbar spines have been proved to be practical, safe, and effective in the treatment of degenerative disease, trauma, scoliosis, infection and tumor [1]. Iatrogenic complications due to mal-positioning of pedicle screws include nerve root injury, vascular injury, and hollow organ injury [2].

**Fig. 1:**
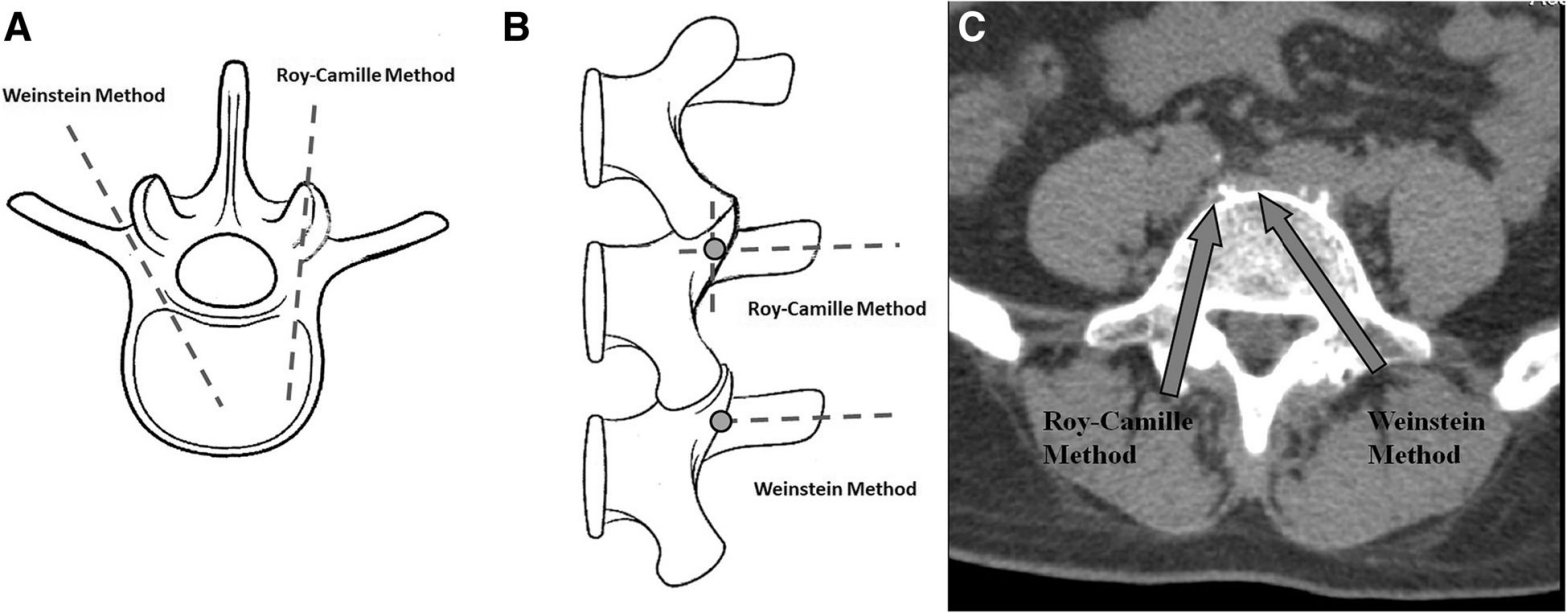
**a**, **b** and **c**. Illustration of entry points of pedicle screws via Roy-Camille or Weinstein method. **a**. Axial view of both methods. **b**. Posterior view of both methods. **c**. Roy-Camille or Weinstein method in axial view of CT scan. The gray arrow indicates the trajectory of pedicle screw placement in lumbar spine

Two common methods clinically used for pedicle screw placement were Roy-Camille [3] and Weinstein [4] methods, which were advocated using different anatomic landmarks (Fig. 1). The Roy-Camille method [3] was advocated at the landmark of medial of the accessory process and 1 mm below the facet joint. The Weinstein method [4] was advocated at the lateral and inferior corner of the superior articular face. The Weinstein method for pedicle screw fixation is commonly used in the Wiltse approach with paraspinal muscle-sparing [5]. Therefore, it is important for spine surgeons to be familiar with the detailed anatomy of the pedicle orientation in relation to the morphology of each vertebra so as to avoid iatrogenic complications during pedicle screw placement.

Morphometric studies have been performed using direct measurements on cadavers or radiologic measurements in CT scans among different Caucasian [6–8], Indian [9, 10], Korean [11] and Chinese [12] populations. Anatomic differences and variations among Caucasian and Taiwanese populations have also been reported [13].

This study analyzed the lumbar morphology and pedicle orientation at L1 to L5 in Taiwanese population. First, pedicle orientation related to the identical lumbar morphology was defined to facilitate the anatomy during pedicle screw placement with either Roy-Camille or Weinstein method. Second, anatomic differences between Caucasian and Taiwanese populations regarding lumbar morphology and pedicle orientation were explored.

## Methods

The research protocol was approved by the institutional review board of Taipei Veterans General Hospital (2017–10-008 BC). Young patients, aged 20–50 years, who were arranged for CT scan examination due to persisted back pain and sciatica were studied. In other words, the study group was not normal distribution of the Taiwanese population. Computed tomography scans (Toshiba, Aquilion, Tokyo, Japan) of the lumbar spines with 3-mm cut plane were performed and reviewed using a computer software for measurement (Smart viewer 3.2; Taiwan Electronic Data Processing Cooperation, Taipei, Taiwan). Moreover, the measurement plane was defined at the mid-pedicle isthmus in the axial plane, which was determined at the sagittal view.

The inclusion criteria for patients with each lumbar vertebra to be measured were as follows: (1) no previous surgery, (2) no vertebral pathology such as infection, tumor and fracture, (3) no severe degeneration, (4) symmetric pedicle distribution on the axial planes measured, and (5) the cut plane parallel to the inferior endplate. A total of 90 patients met the inclusion criteria for further measurements from January 2014 to September 2014. This number was chosen arbitrarily without a prior power analysis.

There were 52 males and 38 females with mean age at 34.5 years (range, 22 to 46 years). To avoid bias, all radiologic measurements were performed on the radiographs by the same investigator (H.H.L.) to ensure consistent results [14]. So we did not check the inter- or intra-observer bias before all measurements. Three measurements were obtained with their arithmetic mean calculated as data for analysis.

Before measuring each vertebra at different levels, a tangential line was drawn along the bilateral transverse process and another perpendicular midline axis (AP axis) which bisected the vertebral body. The measurement plane was defined at the mid-pedicle isthmus in the axial plane, which was determined at the sagittal view.

**Fig. 2:**
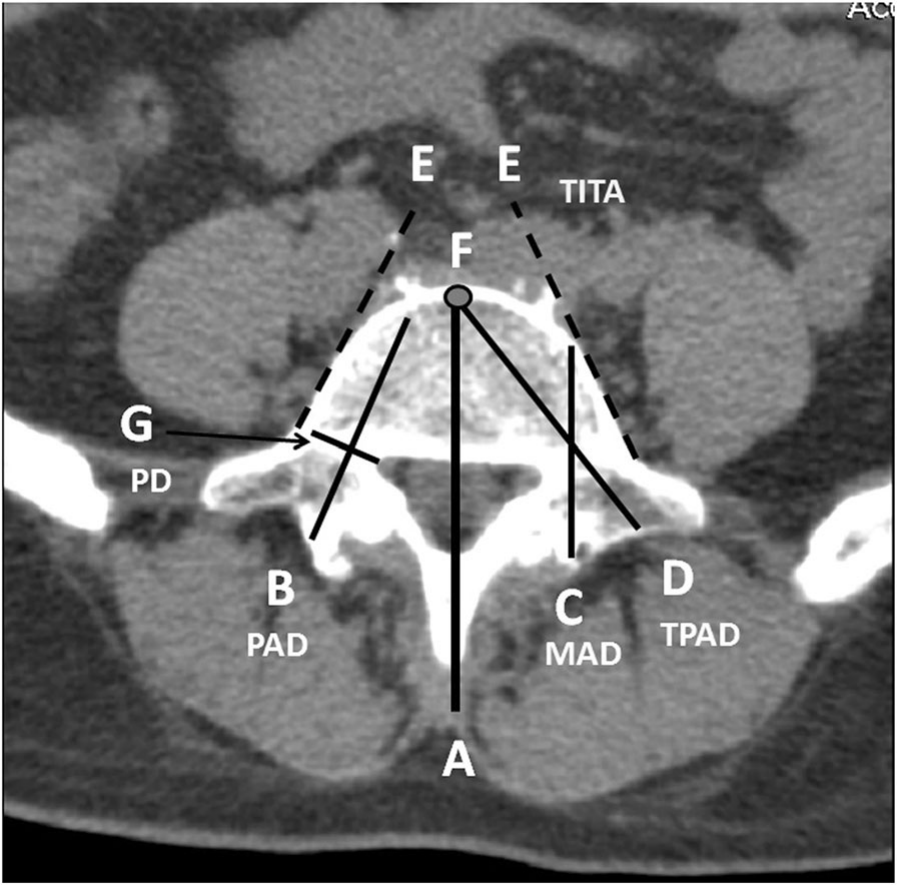
**a** to **g**. Illustration of various radiologic parameters for measurement. **a**. Antero-posterior (AP) midline axis. **b**. Distance to anterior cortex along pedicle axis (pedicle axis distance) (PAD). **c**. Distance to anterior cortex along AP midline axis (midline axis distance) (MAD).**d**. Distance from interface between transverse process and pedicle to anterior cortex of vertebra along pedicle axis (transverse pedicle axis distance) (TPAD). **e**. Lateral tangential lines (dotted lines E) along lateral borders of each vertebra. Transverse intertangential angle (TITA) was defined as the angle between two dotted lines E at each side of the vertebra. **f**. Point F was defined as the anterior border of the vertebra through midline axis and also taken as end point for pedicle screw insertion using the Weinstein method. PA (pedicle angle) was the angle between lines A and B. **g**. Pedicle diameter (PD) was taken as the line perpendicular to line B, which was also taken as pedicle axis distance (PAD)

The pedicle longitudinal axis was marked through the mid-pedicle level, which was defined as pedicle diameter (PD), as adopted by Olsewski et al. [6] (Fig. 2) The distance from the lamina to the anterior cortex along the AP midline axis was defined as the midline axis distance (MAD), as reported by Zindrick et al. [15] (Fig. 2). The parameter regarding the distance from the lamina to the anterior cortex along the longitudinal pedicle axis was defined as pedicle axis distance (PAD), which was also used for measuring the pedicle angle (PA), as adopted by Zindrick [15] and Olsewski et al. [6] (Fig. 2) The PAD was assumed as the pedicle screw length in the Roy-Camille approach.

The anterior border of the vertebrae along the midline axis was selected as the assumed target point for pedicle screw insertion in the Weinstein approach. The distance from this assumed target point to the junction between transverse process and pedicle, which was the entry point for the Weinstein method, was measured. The distance was defined as transverse pedicle axis distance (TPAD) (Fig. 2), which was taken as the pedicle screw length in the Weinstein method.

The pedicle angle (PA) [10, 15], defined as the angle between the AP midline axis and pedicle axis, was measured. Moreover, the transverse intertangential angle (TITA) was defined as the angle between two lateral tangential lines along the lateral borders of each vertebra from posterior to anterior, as adopted by Van Schaik et al. [8] (Fig. 2). A positive value indicates convergence while a negative value implies divergence.

The two sides of the PA, PD, midline and pedicle axis in one vertebra were taken as independent sets of data. However, each vertebral body only had its own identical transverse intertangential angle (TITA). The mean measurements of PA, MAD, PAD and transverse pedicle diameter from L1 to L5 levels were compared with those reported by Zindrick et al. [15] Results of TITA from L3 to L5 levels obtained in this study were compared with those reported by Van Schaik et al. [8]

A total of 2905 pedicle measurements including transverse PA, MAD, PAD and transverse pedicle isthmus widths, as adopted from Zindrick et al. [15], were made from T1 to L5 using spinal CT scans and individual roentgenograms without detailed patient information. Table 1 displays individual sample sizes in each radiographic parameter. The morphology of TITA, as adopted from van Schaik et al. [8] from L3 to L5 levels was studied in 71 patients with mean age of 41.4 ± 12.1 years (Table 2).

**Table 1:** Radiologic Measurements in Various Parameters at L1 to L5 in Our Results and Zindrick et al

Radiologic parameters	Study	L1				L2				L3				L4				L5			
		Mean	SD	Range	Number	Mean	SD	Range	Number	Mean	SD	Range	Number	Mean	SD	Range	Number	Mean	SD	Range	Number
Pedicle angle (PA) (^0^)^a^	Our results	9.3	1.6	6–14	156	7.4	2.1	4–12	162	7.9	1.8	4–13	154	11.2	3.2	5–19	156	17.0	3.1	9–25	146
	Zindrick et al	10.9	2.2	6.5–14.5	26	12	3.5	5–17.5	38	14.4	3.8	8.0–23.5	68	17.7	5.2	5.5–27.5	76	29.8	6.3	19–44	76
Midline axis distance (MAD) (mm)^a^	Our results	40.3	3.3	34–49	156	41.1	3.3	36–47	162	38.8	3.6	31–46	154	37.3	5.3	25–45	156	31.4	3.6	25–44	146
	Zindrick et al	44.7	4.5	36–51	26	45.5	3.7	37–52	35	44.4	5.1	31–54	64	40.7	4.3	27–48	74	33.7	5.6	20–44	69
Pedicle axis distance (PAD) (mm)^a^	Our results	44.8	4.8	37–56	156	46.4	5.8	36–58	162	43.5	5.0	35–58	154	43.7	5.0	33–58	156	43.3	4.5	32–57	146
	Zindrick et al	50.7	4.3	42–57	26	51.9	3.7	45–58	35	51.9	4.6	42–62	64	49.7	4.4	43–62	74	51.0	4.7	42–62	69
Pedicle diameter (PD) (mm)^a^	Our results	5.7	1.2	4–10.4	156	6.4	1.6	4–12.2	162	7.6	2.0	4.3–14.2	154	10.1	1.8	7–14.4	156	12.6	2.1	9.6–25.1	146
	Zindrick et al	8.7	2.3	4.5–13	26	8.9	2.2	4–13	30	10.3	2.6	5.3–16	49	12.9	2.1	9.1–17	36	18.0	4.1	9.1–29	56
Transverse pedicle axis distance (TPAD) (mm)	Our results	45.3	3.5	40–52	156	48.1	3.6	42–55	162	46.1	4.1	40–57	154	45.7	4.1	39–55	156	44.1	4.3	35–57	146
	Zindrick et al																				

No TPAD results were revealed in the Zindrick et al. These columns were empty

The two sides of the pedicle angle, pedicle diameter, midline and pedicle axis and TPAD in one vertebrae were considered as independent sets of data

^a^ meant statistically significant between Caucasian and Taiwanese populations

**Table 2:** Transverse Intertangential Angle (TITA) Between Our Results and Van Schaik et al.

	L1			L2			L3 *			L4*			L5*		
	Mean	SD	Range	Mean	SD	Range	Mean	SD	Range	Mean	SD	Range	Mean	SD	Range
Our results	− 13.4	7.6	− 23 to + 1	− 16	8.3	− 29 to − 5	− 9.7	7.5	+ 5 to + 29	2.4	8.5	+ 4 to + 21	44.8	13.1	+ 16 to + 78
Van Schaik et al							− 14.3	12	− 44 to + 12	6.3	11.5	− 24 to + 37	53	15.4	+ 18 to + 88

TITA is the angle between two lateral tangential lines along the lateral borders of each vertebrae was also measured from posterior-anterior axis

Positive value defined as convergence, and negative value defined as divergence from posterior-to-anterior axis

Van Schaik et al. revealed his results in L3 to L5 only without L1 and L2 results provided

* meant statistically significant between Caucasian and Taiwanese populations

Statistical analysis was performed with the SPSS for Windows statistical package, version 15.0 (SPSS, Chicago, Illinois). Independent-samples *t* test was employed to compare mean data between Taiwanese and Caucasian subjects. A *P* < 0.05 was considered statistically significant (The significance level was set at *P* = 0.05). G*Power software (Heinrich-Heine Universit¨at D¨usseldorf, D¨usseldorf, Germany) was utilized to calculate the power in each comparison between two groups.

## Results

According to the inclusion criteria, 78 patients were selected for L1, 81 for L2, 77 for L3, 78 for L4 and 73 for L5 measurements. Significant differences (P < 0.05) in PA, PD, MAD and PAD at L1 to L5 levels were observed between Caucasian and Taiwanese populations (Table 1). Moreover, significant difference (P < 0.05) in TITA from L3 to L5 levels was also found between Caucasian and Taiwanese populations (Table 2). Van Schaik et al. [8] reported only results at L3 to L5 levels. All results obtained in this study were presented in detail in Tables 1 and 2.

As seen in Table 1, the mean PA from L1 to L5 were 9.3^0^ ± 1.6^0^, 7.4^0^ ± 2.1^0^, 7.9^0^ ± 1.8^0^, 11.2^0^ ± 3.2^0^, 17.0^0^ ± 3.1^0^, respectively; with the largest PA found at L5 level (range, 9^0^–25^0^). As shown in Table 2, the mean TITA from L1 to L5 were − 12.6^0^ ± 8.9^0^, − 16.4^0^ ± 8.7^0^, − 10.1^0^ ± 8.1^0^, 1.0^0^ ± 10.3^0^ and 43.7^0^ ± 14.3^0^, respectively; revealing the lower the vertebra, the greater the convergent angle along the pedicle lateral border from L3 to L5.

The mean PAD from L1 to L5 were 44.8 ± 4.8 mm, 46.4 ± 5.8 mm, 43.5 ± 5.0 mm, 43.7 ± 5.0 mm and 43.3 ± 4.5 mm, respectively; indicating the more caudal the lumbar vertebra, the shorter the PAD.

The mean MAD from L1 to L5 were 40.3 ± 3.3 mm, 41.1 ± 3.3 mm, 38.8 ± 3.6 mm, 37.3 ± 5.3 mm and 31.4 ± 3.6 mm, respectively; with L2 having the longest MAD (range, 36–47 mm) and L5 having the shortest (range, 25–44 mm).

The mean TPAD from L1 to L5 were 45.3 ± 3.5 mm, 48.1 ± 3.6 mm, 46.1 ± 4.1 mm, 45.7 ± 4.1 mm and 44.1 ± 4.3 mm, respectively; with L2 having the longest TPAD (range, 42–55), and L5 having the shortest (range, 35–57 mm).

The present results showed that L5 had the most convergent pedicle axis and shortest body distance in the anterior-posterior (AP) axis and the widest transverse pedicle diameter. L2 had the largest MAD, PAD and TPAD. The reason to explain the result is that L1 is the transitional vertebra at the thoracolumbar junction, which may have some intermediate anatomic characteristics and features. Moreover, the lower caudal lumbar vertebra had more convergent angle of PA and TITA and wider PD from L3 to L5.

Regarding power analysis, G*Power reached 0.99 for the parameters of PA, MAD, PAD and PD at L1 to L5. G*Power exceeded 0.8 for TITA at L4 and L5 (power = 0.84 and 0.85, respectively). However, G*Power for TITA at L3 (power = 0.7) was insufficient to reach a conclusion Based on the G*power software, the parameters were set up as follows: α = 0.05, equal sample sizes (N2/N1 = 1), power = 0.8 and two tails in the formula. With the given results between Taiwanese (mean:-10.1, SD: 8.1) and Caucasian populations (mean:-14.3, SD:12), the recommended sample size for TITA at L3 was 95.

## Discussion

This study observed difference in lumbar vertebrae morphology including PA, (PD, MAD and PAD at L1 to L5 levels between Caucasian and Taiwanese subjects. Moreover, racial differences were also found in TITA from L3 to L5 levels between Caucasian and Taiwanese populations. Only raw data after direct measurement from CT scan without age normal distribution were shown, and the number was arbitrarily chosen without prior power analysis.

There are some major weaknesses or drawbacks in this study. First, the patients did not select at the normal age distribution, which may not substantially reflect the anatomic distribution due to selection bias. Second, patients’ demographic data regarding height and body weight, which may also play roles in anatomic variation, were not included in the analysis. Third, the data from both pedicles in the same vertebrae were taken as independent. Given normal symmetry, this assumption may be incorrect and thus the importance of the differences may be overestimated. Fourth, intra- and inter-observer reliability were not examined, neglecting approximately 5^0^ to 7^0^ in inter-observer variability [16]. Moreover, some assumptions may not be right, such as the PAD taken as pedicle screw length and PA taken as the trajectory angle in the Roy-Camille method. Moreover, the enrolled patients were too few to reach conclusions with adequate prior power analysis. Finally, performing post-hoc power analysis to show the adequate sample sizes was also a limitation of the study.

Pedicle angle (PA) from L1 to L5 was significantly different from those reported by Zindrick et al. [15]. In other Caucasian studies reported by Olsewski et al. [6] and Marchesi et al. [17], racial difference in PA from L1 to L5 levels as measured on radiographs was also found regardless of gender. Similar trends were observed in Caucasian and Taiwanese populations that the more caudal vertebra had larger PA with L5 having the largest. PA could be taken as the trajectory angle for pedicle screw placement in the Roy-Camille method. Apparently, surgeons performing the Weinstein approach with free-hand technique would prefer more convergent angle with larger PA. In pedicle screw placement, surgeons had to keep pedicle screws within the pedicle to achieve maximum fixation and pullout-resisting strength and avoid iatrogenic complications regardless of the method adopted.

Regarding TITA, the more caudal vertebra had more convergent pedicle axis with L5 having the largest TITA, as seen in the results of this study and Zindrick et al. [8] L5 had the largest PA and TITA but shortest MAD, PAD and TPAD. Consequently, L5 vertebra has more hemispherical morphology with more convergent pedicle axis from the posterior-anterior axis [18, 19] in relation to the L5 vertebral body. Hence, it is easy to break through the anterior cortex when adopting the Roy-Camille method for L5 pedicle screw placement due to the shorter AP axis and greater convergence along the pedicle axis. Degenerative spondylolisthesis occurs most commonly at the L4-L5 level [20], and surgeons have to keep in mind the unique morphology of L5 when placing pedicle screws with either method via free-hand technique.

Regarding pedicle axis distance (PAD) and midline axis distance (MAD), the present study found that L2 had the largest PAD and MAD, which is consistent with the finding of Zindrick et al. [15]. Among the Caucasian population, Olsewski et al. [6] reported that L4 had the largest posterior-to-anterior distance. Either PAD or MAD may be taken as a reference for the pedicle screw length when performing the Roy-Camille method according to the surgeons’ preferences with different trajectory angles. Increased screw length could enhance fixation strength of the pedicle screw within the bone [21]. However, iatrogenic complication could occur if the screws penetrate into the abdominal cavity.

No matter which method was applied for pedicle screw placement, surgeons should carefully use the guide pin and pedicle finder to check the tract length and its convergence with repeated checks using portable C-arm images. Proper length preparation in the vertebral body could avoid anterior penetration into the abdominal cavity so as to avoid complications including vascular injury and hallow organ perforation. Hirano et al. [22] pointed out that the pedicle of spine plays a more important role in resisting pullout strength than the vertebrae. Li et al. [23] reported an approximate 23.4% decrease in pullout strength of pedicle screws under lateral wall perforation. Therefore, surgeons had to make sure that the screw is within the pedicle without medial or anterior or lateral wall perforation when performing either method although some surgeons may adopt the “in-out-in” technique for pedicle screw placement.

Pedicle diameter (PD) may be used as a reference for surgeons when choosing the pedicle screw diameter. This study and Zindrick et al. [15] found that L5 had the largest PD, and the more caudal lumbar vertebra had larger PD. Similar results were also observed among Caucasians as reported by Olsewski et al. [6] Wittenberg et al. [24] reported that pedicle screw of larger diameter increased axial pullout force and enhanced spinal stability, but might have higher incidence of breaking through the pedicle medial wall and further damage nerve root, especially with inappropriate convergent angle. Misenhimer et al. [25] indicated that the ideal screw diameter was 80% of PD. According to the present results, the ideal pedicle screw diameter from L1 to L5 were 4.7 mm, 5.9 mm, 6 mm, 8.1 mm and 12.1 mm, respectively. Hence, the diameter of 6.0 mm could be safely used in the Taiwanese population with both methods for lower lumbar pedicle screw fixation (L3 to L5).

Owing to individualized variability among patients and populations, surgeons need to be aware of the possible lumbar morphology and pedicle orientation when placing pedicle screws with either the Roy-Camille or Weinstein method using free-hand technique or guided by navigation system. Meticulous pre-operative planning with CT scan is important to ensure appropriate diameter, length and trajectory for pedicle screw placement.

The results obtained illustrated variability of each lumbar morphology and pedicle orientation and differences between Caucasian and Taiwanese populations. Such information may help surgeons place the lumbar pedicle screws more safely and accurately to avoid iatrogenic complications. Moreover, meticulous pre-operative planning and intra-operative multiple portable C-arm checkup, guide pin with pedicle finder systems could also be helpful to alleviate iatrogenic complications if the O-arm navigation system is not routinely used in clinics.

## Abbreviations

MAD: Midline axis distance
PA: Pedicle angle
PAD: Pedicle axis distances
PD: Pedicle diameter
TITA: Transverse intertangential angle
TPAD: Transverse pedicle axis distance
